# Self-reported confidence in patient safety competencies among Chinese nursing students: a multi-site cross-sectional survey

**DOI:** 10.1186/s12909-020-1945-8

**Published:** 2020-01-31

**Authors:** Fei Fei Huang, Xiao Ying Shen, Xue Lei Chen, Li Ping He, Su Fen Huang, Jin Xiu Li

**Affiliations:** 1 School of Nursing, Fujian Medical University, No 1 Xuefu north Road, Minhou county, Fuzhou, 350108 Fujian China; 2 School of Nursing, Harbin Medical University, the 2nd Affiliated Hospital of Harbin Medical University, Herbin, China; 3 School of Nursing, Guilin Medical University, Gulin, China; 4 School of Nursing, Changzhi Medical College, Changzhi, China; 5 School of Nursing, Quanzhou Medical College, Quanzhou, China; 6 School of Nursing, Ji Shou University, Jishou, China

**Keywords:** Patient safety, Nursing, Survey, Undergraduate students

## Abstract

**Background:**

Nursing interns are an important backup force for nursing professionals, so efforts to strengthen their patient safety (PS) competencies are a major priority. To do so requires assessing the strengths and weaknesses of Chinese nursing students’ PS competence and identifying the influencing factors.

**Methods:**

This was a multi-site, cross-sectional, web-based study that was carried out between September 2018 and January 2019. A national online survey was completed by 732 Chinese undergraduate nursing students. Our primary outcome factor was the Health Professional Education in Patient Safety Survey score. We also collected socio-demographic and clinical practice-related characteristics as independent variables.

Multiple stepwise linear regression was performed to identify predictors of PS competence.

**Results:**

Chinese undergraduate nursing students were fairly confident in their clinical safety skills but less confident in what they learned about sociocultural or context-dependent aspects of PS and speaking up about PS, including effective communication and understanding human and environmental factors. Less than half of the students felt that they could approach someone engaging in unsafe practice and were reluctant to voice concern about adverse events. We observed significant differences in PS competence between students from different regions, across different PS learning styles (self-study and classroom theoretical study), with different self-assessed PS competence levels, and with experiences of adverse events (*p* < 0.05). These factors accounted for almost 15% of the total variance in PS competence scores (adjusted *R*^*2*^ = 0.15, *p* = 0.00).

**Conclusions:**

The results of this study provide a better understanding of PS competence among final-year nursing students in China. Our findings may help nursing educators or healthcare organizations to cultivate and improve PS competence by establishing documented policies or by improving the efficacy of intervention.

## Background

Patient safety (PS) remains under constant scrutiny due to the possibility of preventable harm occurring during medical care [1]. In Chinese hospitals, 40% of patients experience at least one preventable adverse event; this is worrying because such events can increase the risk of mortality and damage of hospital reputation [2]. It is therefore important that healthcare providers are sufficiently knowledgeable to recognize potential safety risks and protect patients from potential harm or avoidable injuries [3]. Nurses represent the front line of care and the largest group of healthcare providers; consequently, they play a vital role in improving the safety and quality of patient care [4].

Nursing students serve as the important reserve force of healthcare providers. Emphasis that efforts to help nursing students reflect on their PS knowledge and competence may prepare them to offer appropriate and safe care in a variety of clinical settings and circumstances [5]. The first step towards this goal is assessing the strengths and weaknesses of nursing students’ PS competence [3, 6]. The PS competence entails six domains: contributing to patient safety culture, working in teams, communicating effectively, managing safety risks, optimizing human and environmental factors, and recognizing and responding to adverse events. Previous studies have explored the PS knowledge and skills of students such as in nursing, pharmacy, and traditional medicine [1,3,6,] and new graduate healthcare professionals [7–9] but limited information is available for final-year nursing students or interns, who are the most likely to commit medical errors [1]. It is estimated that 17 to 53.2% of Chinese nursing interns directly or indirectly cause one or more adverse events [10, 11]. In previous studies, nursing students have reported moderate levels of confidence in most domains of PS competence [3, 6, 12]. However, the evidence for PS competence varies according to the academic year of study, some publications report that confidence in PS competence declines as students are increasingly exposed to the clinical environment [3, 12], while others reported findings that directly opposed to those opinion [13]. More studies are therefore needed to better understand the PS competence of this vulnerable body of nursing students.

Notably, the PS competence acquired by nursing students in the classroom is lower than that gained in the clinical setting [3, 8]. For example, undergraduate nursing students in Australia reported greater confidence in their clinical safety skills as opposed to their skills learned in the classroom, although their confidence regarding teamwork skills and managing safety risks was reduced in the clinical setting [3]. The specific domains of PS competence showing discrepancies between the classroom and clinic are heterogeneous when compared across different countries; this may be due to differences in PS cultures [8]. However, the existing evidence was mostly collected in developed countries [3, 8], with more limited datasets from developing countries (e.g., Korea, Saudi Arabia and Jordan) [1, 13–15]. To our knowledge, little is known about the development of nursing students’ PS knowledge and confidence in China, where PS strategies and education tend to lag behind those in developed countries [16].

There is increasing evidence that more emphasis is being placed on explicit PS competence for undergraduate nursing education and learning [3]. Age, gender, region, academic year, and employment status were previously found to be associated with the PS competence of nursing students [9, 13]. However, these findings were obtained using univariate inferential statistical analyses [15]; confirmatory regression analysis is still required. In addition, knowledge and experience are known to underpin PS competence [7]. Hospital rank,^1^ educational background, experiences of adverse events and reporting behavior may also influence the PS competence of nursing students.

The purposes of this nationwide cross-sectional study were to: (a) describe and compare Chinese undergraduate nursing students’ perception of confidence in PS knowledge and competence acquired in both classroom and clinical settings, (b) describe nursing students’ confidence relating to broader aspects of PS and speaking up about PS, and (c) explore the factors that might influence nursing students’ PS competence.

## Methods

### Study design

This was a multi-site, cross-sectional, web-based study that was performed between September 2018 and January 2019. The study conformed with the Strengthening the Reporting of Observational studies in Epidemiology (STROBE) statement [17].

### Setting and participants

Seven four-year nursing Bachelor Program of Chinese universities were invited to participate as research partners. To obtain a representative sample, the universities were selected from seven Chinese administrative provincial regions and were representative of the population density, economic development, and medical services of their respective regions. The seven provincial regions were as follows: North (Shanxi), East (Fujian), Northeast (Heilongjiang), Central (Hunan), Southwest (Guizhou), South (Hainan), and Northwest (Xinjiang) China. In addition, one to two nursing faculty members at each participating university agreed to be a research partner and act as a point of contact at their university. Undergraduate nursing students at each of the seven participating universities who were (a) undertaking final-year clinical internship practice and (b) willing to participate in this study were eligible. According to Pett et al. (2003) [18], 10–15 participants per item were considered appropriate for a target sample size. Considering a potential non-response rate of 15%, the final target sample size was 770. That is, at each university, 110 eligible participants were recruited by convenience sampling methods.

### Measures

The main outcome factor in our study was the Health Professional Education in Patient Safety Survey (H-PEPSS), which was used to measure the self-reported PS competence of health professionals and students [19]. The H-PEPSS has been translated into Chinese and its reliability and validity have been confirmed [20]. The Chinese version of H-PEPSS (H-PEPSS-CV) consists of 32 items divided into three sections, as follows.

- Section 1: “Students’ knowledge about specific patient safety content areas.” This section features two versions of classroom and clinical practice. Each version includes 20 items covering clinical safety issues (4 items) and six dimensions of the Safety Competencies Framework (Ginsburg et al., 2012): communicating effectively (3 items); working in a team with other health professionals (3 items); managing safety risks (3 items); understanding human and environmental factors (2 items); recognizing, responding to, and disclosing adverse events and close calls (2 items); and the culture of safety (3 items).
- Section 2: “How broader patient safety issues are addressed in health professional education.” (7 items).
- Section 3: “How able and comfortable the participants are speaking up about patient safety issues.” (5 items).

Each section is completed by adding together the students’ responses to each item. For item scoring, responses range from “strongly disagree” (scored as 1) to “strongly agree” (scored as 5). Higher domain scores or total scores represent higher levels of students’ perceived PS competence. The scale has achieved a Cronbach’s α of 0.95 and 2-week test-retest reliability of 0.88.

The socio-demographic and clinical practice-related characteristics as independent variables were also collected, including age, gender, only-child status, hospital rank, whether the participant had previously received PS education, whether the participant had experienced adverse events, whether they had disclosed medical errors, and their PS learning method (self-study, theoretical study in the classroom or clinical training).

### Data collection

An online questionnaire (hosted by Wenjuanxing, http://www.wjx.cn, a popular online survey platform in China) was made available to all eligible nursing students at each participating university. Before survey, all participants were obtained by written informed consent. To encourage participation, all nursing students at these seven universities were told that this web-based survey was voluntary, anonymous, and confidential, and also that 80% of them would randomly receive a bonus (named as “Hongbao” in Chinese) (containing 10 yuan) after they completed the questionnaire.

### Data analysis

SPSS 24.0 software (SPSS Inc., Chicago, IL) was used to perform descriptive and inferential statistical analyses. Missing data were replaced using mean value substitution, and *p* < 0.05 was considered to be statistically significant. Socio-demographic and clinical practice-related data were summarized using descriptive statistics. Mean (± standard deviation) PS scores for each domain were calculated by averaging the items. Paired t-tests were performed to identify significant differences between classroom and clinical scores. Cohen’s effect size was calculated for statistically significant pairwise comparisons.

Scores relating to broader aspects of PS and speaking up were categorized into strongly agree/agree (4–5), and neutral/disagree/strongly disagree (1–3); the proportion of students who strongly agreed/agreed were reported descriptively. Chi square tests were conducted using categorized outcomes. Independent t-tests and one-way analyses of variance (ANOVAs) were performed to identify differences in PS competence scores across the independent variables. Multiple stepwise linear regression was performed to identify predictors of PS competence; the dependent variable was PS competence score. Variables showing statistical significance in the t-test or one-way ANOVA were then selected as independent variables for subsequent analysis (*p* < 0.05). Categorical variables were recoded into dummy variables for the multiple linear regression analysis.

## Results

A total of 732 valid questionnaires were returned out of the 770 that were distributed (representing a 95.06% response rate). The mean age of students was 21.56 ± 0.96 years. Of the entire cohort, 97.70% of the students were undertaking clinical practice in a grade A tertiary hospital, and 1.10 and 0.50% of the students were working in grade A secondary and grade B tertiary hospitals, respectively. Socio-demographic and educational characteristics of the nursing students involved in this study are shown in Table 1.

**Table 1:** Socio-demographic characteristics of participants (*n* = 732)

Variable	n (%)	In the classroom		In the clinical practice		Broader aspects of patient safety		Comfort in speaking up about patient safety.	
		Total scores	*t* /F value	Total scores	*t* /F value	Total scores	*t* /F value	Total scores	*t* /F value
Gender									
Male	60(8.20)	78.62 ± 12.69	−0.34	78.42 ± 14.08	−0.07	26.52 ± 4.97	−0.10	17.58 ± 3.50	0.11
Female	672(91.80)	79.11 ± 10.43		78.52 ± 10.44		26.57 ± 4.33		17.54 ± 3.09	
The only-child									
Yes	143(19.60)	81.75 ± 10.63	3.39*	81.47 ± 11.36	3.70*	27.50 ± 4.57	2.73*	17.84 ± 3.12	1.28
No	589(80.40)	78.41 ± 10.53		77.79 ± 10.52		26.34 ± 4.31		17.47 ± 3.12	
Prior experience of patient safety education									
Yes	583(79.70)	80.14 ± 10.25	5.52*	79.50 ± 10.41	4.99*	26.95 ± 4.17	4.76*	17.54 ± 3.18	−0.03
No	149(20.30)	74.85 ± 11.07		74.62 ± 11.33		25.06 ± 4.87		17.55 ± 2.92	
The safety learning methods									
Self-study									
No	561(76.60)	78.23 ± 10.67	−3.90*	77.76 ± 10.85	−3.43*	26.24 ± 4.34	−3.75*	17.39 ± 3.02	−2.32*
Yes	171(23.40)	81.81 ± 10.04		80.96 ± 10.20		27.66 ± 4.37		18.02 ± 3.42	
Theoretical study in Classroom									
Yes	526(71.90)	76.24 ± 10.55	−4.55*	75.76 ± 10.83	−4.36*	25.40 ± 4.82	−4.24*	17.45 ± 3.13	−.465
No	206(28.10)	80.17 ± 10.46		79.58 ± 10.57		27.02 ± 4.12		17.57 ± 3.12	
Clinical training									
Yes	470(64.20)	77.12 ± 10.83	−3.72*	76.88 ± 11.13	−3.06*	25.74 ± 4.84	−3.85*	17.36 ± 3.09	−1.13
No	262(35.80)	80.15 ± 10.37		79.41 ± 10.48		27.03 ± 4.04		17.64 ± 3.14	
Experience of adverse events									
Yes	156(21.30)	76.90 ± 10.96	−2.95*	76.52 ± 11.12	−2.68*	25.75 ± 4.23	−2.88*	17.55 ± 2.81	−0.15
No	576(78.70)	79.72 ± 10.38		79.11 ± 10.55		26.85 ± 4.25		17.59 ± 3.12	
Disclosing behavior									
Yes	690(94.30)	79.64 ± 10.48	2.12*	79.06 ± 10.66	1.64	26.90 ± 4.10	1.25	17.65 ± 3.02	−0.39
No	42(5.60)	75.74 ± 12.24		75.98 ± 12.41		25.74 ± 5.40		17.86 ± 3.19	
Self-assessment patient safety competence									
Very well	66(9.00)	81.71 ± 13.32	14.96*	81.60 ± 13.60	13.20*	27.95 ± 5.61	8.43*	18.31 ± 3.84	1.13
Well	293(40.00)	81.96 ± 10.23		81.26 ± 10.49		27.51 ± 4.43		17.49 ± 3.54	
moderate	348(47.50)	76.77 ± 9.21		76.22 ± 9.56		25.64 ± 3.71		17.41 ± 2.61	
Poor	21(2.90)	73.10 ± 11.48		72.71 ± 8.91		25.24 ± 4.39		17.86 ± 2.29	
Very poor	4(0.50)	55.50 ± 16.34		58.25 ± 12.82		23.00 ± 11.05		18.25 ± 2.36	
Regions									
Fujian	161(22.00)	76.03 ± 10.89	5.80*	75.33 ± 11.14	7.25*	24.61 ± 4.96	11.96*	16.60 ± 3.02	3.96*
Guangxi	76(10.40)	76.84 ± 9.01		74.93 ± 8.71		25.26 ± 3.39		17.49 ± 2.79	
Guizhou	89(12.20)	78.66 ± 9.29		78.20 ± 9.72		26.98 ± 3.99		18.01 ± 3.27	
Heilongjian	125(17.10)	82.02 ± 11.29		82.06 ± 11.07		28.26 ± 4.32		18.07 ± 3.32	
Hunan	114(15.60)	80.57 ± 9.23		80.30 ± 9.11		27.04 ± 3.39		17.79 ± 2.51	
Shanxi	106(14.50)	78.80 ± 9.41		78.93 ± 10.10		26.87 ± 3.96		17.39 ± 2.82	
Xinjiang	61(8.40)	82.14 ± 13.86		80.51 ± 13.59		27.95 ± 4.70		18.12 ± 4.13	

*p < 0.01

### Confidence in PS dimensions in classroom and clinical settings

As shown in Table 2, the mean scores were all above 3.5 (out of 5) for PS dimensions and individual items in the classroom and clinical settings. At the dimension level, nursing students were most confident in their learning of “clinical safety skills” in the classroom (4.1 ± 0.58) and “managing safety risks” in the clinical setting (4.00 ± 0.59). They were least confident in their learning of “understanding human and environmental factors” in the classroom (3.79 ± 0.70) and “communicating effectively” in the clinical setting (3.77 ± 0.65). The proportion of nursing students who were confident about what they were learning with regards to each PS dimension (based on responding “agree” or “strongly agree”) ranged from 68.25 to 84.47% for classroom learning, and from 67.40 to 81.43% for learning in the clinical setting.

**Table 2:** Classroom and clinical self-reported patient safety domains scores for undergraduate nursing students (*n* = 732)

Patient safety areas	Setting	Mean (SD)	Effect size	Paired *t-*test (*t* and *p* value)	Agree/strongly agree		
					N	%	χ2 2-tail *p* value
Culture of safety	Classroom	3.91(0.65)	−0.02	*t* = −0.96 *p* = 0.34	558	76.17	χ2 = 0.19 *p* = 0.66
	Clinical	3.94(0.68)			565	77.17	
Work in teams with other health professionals	Classroom	4.1(0.60)	0.14	*t* = 9.34 *p* = 0.00	618	84.47	χ2 = 13.70 *p* = 0.00
	Clinical	3.93(0.61)			562	76.8	
Communicating effectively	Classroom	3.92(0.66)	0.11	*t* = 7.41 *p* = 0.00	549	74.93	χ2 = 10.44 *p* = 0.00
	Clinical	3.77(0.65)			493	67.40	
Managing safety risks	Classroom	3.82(0.67)	−0.14	*t* = −8.54 *p* = 0.00	508	69.40	χ2 = 28.53 *p* = 0.00
	Clinical	4.00(0.59)			596	81.43	
Understanding human & environmental factors	Classroom	3.79(0.70)	−0.10	*t* = −5.95 *p* = 0.00	500	68.25	χ2 = 15.03 *p* = 0.00
	Clinical	3.92(0.63)			566	77.3	
Recognize & respond to remove immediate risks	Classroom	3.90(0.66)	−0.02	*t* = −1.19 *p* = 0.23	558	76.20	χ2 = 1.42 *p* = 0.43
	Clinical	3.93(0.63)			577	78.80	
Clinical safety skills	Classroom	4.11(0.58)	0.11	*t* = 6.84 *p* = 0.00	612	83.63	χ2 = 2.66 *p* = 0.06
	Clinical	3.98(0.60)			588	80.33	
Total score	Classroom	4.94(0.66)	0.02	*t* = 2.61 *p* = 0.00	557	76.15	χ2 = 0.19 *p* = 0.66
	Clinical	4.91(0.67)			564	77.03	

There are significantly statistical difference between classroom learning and learning in the clinical setting on mean scores of the majority of PS dimensions, such as “clinical safety skills,” “working in teams with other health professionals,” and “communicating effectively” (all *p* < 0.05). All of these statistically significant differences in PS dimensions had small effect sizes and are therefore likely to be of low clinical significance. There were no significant differences in the remaining PS dimensions, such as the culture of safety.

The trend for different learning confidence levels between the classroom and clinical setting was further explored by examining specific items. Except for the “recognizing an adverse event or close call” item, we found statistically significant differences (all with small effect sizes) for all the PS items between the classroom and clinical setting. Furthermore, we identified significant correlations between the students’ scores for the classroom and clinical setting (*p* = 0.00) for most items (Table 3).

**Table 3:** Descriptive statistics of each H-PEPSS-CV item (*n* = 732)

Patient safety areas	Setting	Mean (SD)	Effect size	Paired *t-*test (*t* and *p* value)	Agree/strongly agree		
					N	%	χ2 2-tail *p* value
T1 Safe clinical practice in general	Classroom	3.76(0.82)	−0.07	*t* = −3.79	510	69.70	χ2 = 8.68
	Clinical	3.87(0.77)		*p* = 0.00	560	76.50	*p* = 0.00
T2 Hand hygiene	Classroom	4.00(0.68)	−0.07	*t* = − 3.79	607	82.90	χ2 = 0.99
	Clinical	4.10(0.70)		*p* = 0.00	621	84.90	*p* = 0.32
T3 Infection control	Classroom	4.28(0.81)	0.27	*t* = 14.49	657	89.80	χ2 = 57.45
	Clinical	3.84(0.78)		*p* = 0.00	546	74.60	*p* = 0.00
T4 Safe medication practices	Classroom	4.40(0.69)	0.21	*t* = 10.72	674	92.10	χ2 = 16.99
	Clinical	4.10(0.69)		*p* = 0.00	624	85.30	*p* = 0.00
T5 The importance of having a questioning attitude and speaking up when you see things that may be unsafe	Classroom	3.80(0.80)	−0.04	*t* = −2.19	515	70.40	χ2 = 1.79
	Clinical	3.86(0.79)		*p* = 0.03	538	73.50	*p* = 0.18
T6 The importance of a supportive environment that encourages patients and providers to speak up when they have safety concerns	Classroom	3.99(0.76)	−0.05	*t* = −2.75	580	79.30	χ2 = 4.04
	Clinical	4.07(0.74)		*p* = 0.01	610	83.40	*p* = 0.04
T7 The nature of systems and system failures and their role in adverse events	Classroom	3.95(0.79)	0.04	*t* = 1.90	577	78.80	χ2 = 3.67
	Clinical	3.88(1.06)		*p* = 0.06	546	74.60	*p* = 0.06
T8 Managing inter-professional conflict	Classroom	4.18(0.71)	0.09	*t* = 5.13	641	87.60	χ2 = 4.07
	Clinical	4.05(0.69)		*p* = 0.00	614	83.90	*p* = 0.04
T9 Sharing authority, leadership and decision-making	Classroom	3.95(0.78)	0.11	*t* = 6.12	572	78.20	χ2 = 14.46
	Clinical	3.78(0.78)		*p* = 0.00	508	69.40	*p* = 0.00
T10 Encouraging team members to speak up, question, challenge, advocate, and be accountable as appropriate to address safety issues	Classroom	4.18(0.70)	0.16	*t* = 9.09	641	87.60	χ2 = 27.81
	Clinical	3.95(0.73)		*p* = 0.00	564	77.10	*p* = 0.00
T11 Enhancing patient safety through clear and consistent communication with patients	Classroom	3.88(0.82)	0.15	*t* = 8.85	547	74.70	χ2 = 40.11
	Clinical	3.63(0.82)		*p* = 0.00	433	59.10	*p* = 0.00
T12 Enhancing patient safety through effective communication with other healthcare providers	Classroom	4.07(0.76)	0.19	*t* = 10.11	596	81.40	χ2 = 50.19
	Clinical	3.77(0.80)		*p* = 0.00	476	65.00	*p* = 0.00
T13 Effective verbal and nonverbal communication abilities to prevent adverse events	Classroom	3.80(0.82)	−0.06	*t* = −3.62	503	68.70	χ2 = 16.69
	Clinical	3.90(0.73)		*p* = 0.00	572	78.10	*p* = 0.00
T14 Recognizing routine situations in which safety problems may arise	Classroom	3.98(0.75)	−0.08	*t* = −4.23	564	77.10	χ2 = 17.87
	Clinical	4.10(0.70)		*p* = 0.00	627	85.60	*p* = 0.00
T15 Identifying and implementing safety solutions	Classroom	3.68(0.81)	−0.12	*t* = −6.33	463	63.30	χ2 = 24.85
	Clinical	3.86(0.74)		*p* = 0.00	551	75.30	*p* = 0.00
T16 Anticipating and managing high risk situations	Classroom	3.80(0.79)	−0.17	*t* = −8.81	496	67.80	χ2 = 48.05
	Clinical	4.05(0.69)		*p* = 0.00	610	83.40	*p* = 0.00
T17 The role of human factors, such as fatigue, which effect patient safety	Classroom	3.70(0.81)	−0.07	*t* = −3.86	465	63.50	χ2 = 12.84
	Clinical	3.81(0.77)		*p* = 0.00	529	72.30	*p* = 0.00
T18 The role of environmental factors such as work flow, ergonomics and resources, which effect patient safety	Classroom	3.86(0.76)	−0.12	*t* = −6.38	534	73.0	χ2 = 18.19
	Clinical	4.03(0.69)		*p* = 0.00	602	82.30	*p* = 0.00
T19 Recognizing an adverse event or close call	Classroom	3.84(0.77)	0.01	*t* = 0.37	541	73.90	χ2 = 0.06
	Clinical	3.83(0.75)		*p* = 0.71	545	74.50	*p* = 0.81
T20 Reducing harm by addressing immediate risks for patients and others involved	Classroom	3.96(0.75)	−0.04	*t* = −2.48	575	78.50	χ2 = 4.80
	Clinical	4.02(0.68)		*p* = 0.01	608	83.10	*p* = 0.03

*Note.* H-PEPSS-CV: the Chinese version of the Health professional education in patient safety survey

### Confidence in knowledge relating to “broader aspects of PS” and “comfort with speaking up”

Nursing students’ self-reported confidence in their learning of “broader aspects of PS” and “comfort with speaking up” was generally lower than their confidence in the seven items related to the “broader aspects of PS” (Table 4). The nursing students most strongly agreed that “reporting can lead to change and improvement” (84.50%),“clinical aspects (e.g., hand hygiene, transferring patients, and medication safety) were well covered in the program” (83.4%), and their “scope of practice is clear” (73.7%). While, other areas pertaining to broader aspects of PS and comfort with speaking up about PS issues had agreement levels below 50%, including “felt they could approach someone engaging in unsafe practice” (44.80%), “consistency in how PS issues are dealt with by different instructors” (46.90%), “it is difficult to question the decisions or actions of those with more authority” (47.00%), and “reporting PS problems will result in negative repercussions for them” (48.80%).

**Table 4:** Broader aspects of patient safety and comfort in speaking up about patient safety

Undergraduate nursing students (*n* = 732)	Mean (SD)	Agree/strong agree	
		N	%
*Broader aspects of patient safety*	3.80(0.63)	520	71.07
As a student, the scope of what was “safe” for me to do in the practice setting was very clear to me	3.86(0.77)	539	73.70
There is consistency in how patient safety issues were dealt with by different preceptors in the clinical setting	3.36(0.97)	343	46.90
I had sufficient opportunity to learn and interact with members of interdisciplinary teams	3.57(0.94)	452	61.80
I gained a solid understanding that reporting adverse events and close calls can lead to change and can reduce reoccurrence of events	4.08(0.73)	619	84.50
Patient safety was well integrated into the overall program	3.80(0.78)	521	71.20
Clinical aspects of patient safety (e.g. hand hygiene, transferring patients, medication safety] were well covered in our program	4.02(0.74)	610	83.40
“System” aspects of patient safety were well covered in our program	3.89(0.76)	556	76.00
*Comfort in speaking up about patient safety*	3.51(0.62)	406	55.50
In clinical settings, discussion around adverse events focuses mainly on system-related issues, rather than focusing on the individual (s) most responsible for the event	3.70(0.83)	477	65.10
In clinical settings, reporting a patient safety problem will result in negative repercussions for the person reporting it	3.30(1.03)	357	48.80
If I see someone engaging in unsafe care practice in the clinical setting, I feel safe to approach them	3.45(0.77)	328	44.80
It is difficult to question the decisions or actions of those with more authority	3.36(0.93)	344	47.00
If I make a serious error, I worry that I will face disciplinary action.	3.76(0.92)	526	71.80

### Factors that influence nursing students’ perceived confidence relating to PS dimensions

Table 1 shows significant differences for various independent variables in confidence relating to PS dimensions learned in the classroom and clinical settings, knowledge of broader aspects of PS, and comfort with speaking up. These independent variables were included in the four corresponding regression models.

Table 5 highlights the four factors that could predict PS competence among Chinese undergraduate nursing students (relating to three models, i.e., for PS competence based on learning in a classroom and in a clinical setting, and knowledge of the broader aspects of PS): (a) region, (b) PS knowledge gained by self-study, (c) PS knowledge gained by theoretical studies in the classroom, (d) self-assessment of PS competence as “moderate or “poor,” and (e) experience of adverse events. These factors accounted for approximately 15% of the total variance (adjusted *R*^*2*^ = 0.15) in PS competence scores. In addition, previous experience of PS education was also a significant influencing factor of PS competence based on learning in a classroom and knowledge of the broader aspects of PS. When investigating students’ comfort with speaking up about PS issues, we found that region and self-study were statistically significant in the univariate analyses, but neither was significant in the final regression model (*p* > 0.05).

**Table 5:** Independent predictors for the level of confidence in PS competence of nursing students (*n* = 732)

Section	Predictors	Reference	*B*	*Standardized beta*	*t*	*p*
The seven PS dimensions in the classroom ^a^	Constant		76.62		27.35	0.00
	Had prior experience of patient safety education	No experience	2.64	0.10	2.58	0.01
	Self-study	Without self-study	2.35	0.09	2.64	0.01
	Theoretical study in Classroom	Without theoretical study	1.86	0.08	2.06	0.04
	Had experience of adverse events	No experience	2.51	0.10	2.78	0.01
	Self-assessment patient safety competence	Very well				
	General		−2.96	−0.14	−2.15	0.03
	Poor		−5.95	−0.09	−2.36	0.02
	Very poor		−19.87	−0.14	−3.87	0.00
	Regions	Shanxi				
	Xinjiang		3.64	0.09	2.28	0.02
The seven PS dimensions in the clinical setting ^b^	Constant		74.53		29.45	0.00
	Self-assessment patient safety competence	Very well				
	General		−3.77	−0.18	− 2.69	0.01
	Poor		−7.33	− 0.11	− 2.88	0.00
	Very poor		−18.50	−0.13	−3.54	0.00
	Regions	Shanxi				
	Fujian		−2.98	−0.12	− 2.36	0.01
	Theoretical study in Classroom	Without theoretical study	2.63	0.11	3.11	0.00
	Had experience of adverse events	No experience	2.20	0.08	2.38	0.02
	Self-study	Without self-study	1.93	0.08	2.12	0.04
Broader aspects of PS	Constant		25.80		22.27	0.00
	Self-assessment patient safety competence	Very well				
	General		−1.41	−0.16	−2.48	0.01
	Regions	Shanxi				
	Heilongjian		1.09	0.09	1.99	0.05
	Fujian		−2.00	−0.19	−3.92	0.00
	Theoretical study in Classroom	Without theoretical study	0.80	0.08	2.16	0.03
	Had experience of adverse events	No experience	1.12	0.10	2.99	0.00
	Had prior experience of patient safety education	No experience	0.88	0.08	2.08	0.04
	Self-study	Without self-study	0.74	0.07	2.02	0.04

^a^*F* = 10.22, *p* = 0.00, adjust *R*^*2*^ = 0.15

^b^*F* = 9.74, *p* = 0.00, adjust *R*^*2*^ = 0.14

^c^*F* = 10.09, *p* = 0.00, adjust *R*^*2*^ = 0.15

## Discussion

To our knowledge, this is the first study to explore self-reported PS competence and influencing factors among undergraduate nursing students in China. Our results revealed moderate self-reported PS competence among Chinese undergraduate nursing students. This level of competence is higher than for nursing students in Jordan [15], Korea [1] and Canada [12], but slightly lower than the overall PS competence of nursing students in Italy [21], Saudi Arabia [13] and Australia [3]. Our finding that nursing students reported higher levels of PS competence for clinical safety compared to the sociocultural aspects of safety is consistent with previous reports relating to nursing and medical students [1, 3, 8].

Although the World Health Organization published a multi-professional PS curriculum guide [22], our findings further reflect the limitations of existing nursing education programs for PS. Similar to United Kingdom, Japan and United states, there is currently no formal PS curriculum for undergraduate nursing programs in China [23]. Instead, PS learning is interspersed with other nursing curricula, and the PS contents overwhelmingly focus on clinical safety issues, with insufficient attention paid to the sociocultural aspects of PS [15].

Similar to previous studies [3, 8, 15], we also observed a difference between nursing students’ confidence with regards to what they learned in the classroom and in clinical settings. This further highlights the current “theory to practice gap” in PS [3, 8]. Nursing students were more confident about what they learned in the classroom compared with what they learned in clinical practice when considering the dimensions of “clinical safety skills,” “communicating effectively about PS issues,” and “working in teams with other health professionals.” Ways to develop clinical safety knowledge, teamwork, and communication, include increasing the application of approaches for the reform of nursing education, including simulation, team-based learning or problem-based learning. However, the confidence of nursing students relating to other intangible dimensions of PS (“managing safety risks” and “understanding human and environmental factors”) were higher for clinical practice compared with the classroom. In mainland China, hospitals rather than nursing schools oversee interns’ nursing safety problems [11]. This implies that a hospital’s culture and clinical practice can promote nursing students’ understanding of the human and environmental factors underpinning PS, and their understanding of how to manage safety risks [14, 24].

As nursing students spend more time in the clinic, they gain greater levels of awareness about gaps in their knowledge [6], such as how to communicate effectively and how to work in teams with other health professionals. Fostering collaboration between nursing faculty and clinical nurses to teach PS content could help close the gap between theory and practice in PS education [6]. Improving the overall integration and implementation of PS issues in the classroom and clinical settings are important issues faced by nursing educators and clinical instructors. In other words, we must understand what is being taught in both settings. This could help address the inconsistencies in how PS issues are dealt with by different instructors, as reported by undergraduate nursing students (only 46.90% in our present study). Consistent with previous research [25], this suggests that preceptor consistency is a real unsolved issue across multiple programs in China, which may influence student accountability and confidence in dealing with errors.

Regarding comfort with speaking up about PS issues, our findings are congruent with previous studies and confirm the persistent theme in PS research, that is, the uncertainty and apprehension around error reporting [6]. Being able to raise PS issues comfortably is persistently dependent on the culture and attitudes in each specific clinical setting. “Safety voice” is a form of discretionary communication and is more likely to be used in corporate culture or supportive environments [26]. The nursing field is characterized by hierarchical power dynamics and rigid role boundaries, which make nursing students less likely to raise concerns [3]. This is supported by our finding that 47% of nursing students comply with unacceptable practices to avoid disrupting their sense of belonging to their nursing team. It is therefore important for nursing educators or clinical instructors to adopt role-modeling behaviors and to guide and encourage nursing students to raise concerns about PS [27]. In addition, they should consider the need for nursing students to freely share and reflect on adverse events/close calls in a not punitive and more constructive environment and in appropriate settings with modern information technology and mobile devices, such as QQ or WeChat.

Notably, this study also revealed that the perceived PS competence of nursing students varied significantly according to region, self-assessment of PS competence, PS training methods (i.e., self-study and theoretical classroom study) and previous experience of PS education or adverse events. Although the *R*^*2*^ values were relatively small, our analyses help to explain the factors that influence PS competence among Chinese undergraduate nursing students; this fills a notable gap in the existing literature. Our findings corroborated those of other multi-center PS studies [1, 3, 13], in that the universities and hospitals where nursing students learn and practice play a key role in influencing the students’ competence in PS. On the other hand, it reflects the status quo of nursing education on safety, that is, few nursing schools in mainland China include a nursing safety or PS curriculum in their teaching program, and the undergraduate interns receive their most knowledge of PS from pre-practice education at teaching hospitals.

This underscores the need to establish a strong and coherent PS curriculum. We also gained a better understanding of the barriers to, and facilitators of, PS competence. Specifically, previous experience of adverse events was an important potential barrier to PS competence, while self-study and theoretical learning about PS issues, and having “very good” self-assessment of PS competence were important facilitators. It is necessary to establish effective teaching and learning strategies that equip nursing students with adequate PS-related knowledge and skills to correctly deal with adverse events if we are to achieve meaningful PS improvements and create harm-free environments for patients [28].

This study has several limitations worth noting. Selection bias is a common limitation of cross-sectional studies as probability sampling is seldom used. Additionally, self-reported data (e.g., adverse events or disclosed medical errors) may have introduced a social desirability bias, which could have led to the over- or under-reporting of PS competence. Further investigations, such as qualitative research or a longitudinal single cohort study, are now required to better understand the confidence of nursing students in their classroom and clinical learning.

## Conclusions

Since nursing interns are a vital backup force for nursing professionals, it is critical to strengthen their competence in PS [29]. These results describe the PS competencies of final-year nursing students approaching entry into professional clinical practice. Overall, Chinese nursing students reported moderately high PS competencies, although socio-cultural aspects were weak. Previous experience of adverse events was identified as a barrier to PS competence, while diversified PS training facilitated PS competence. Our findings may help nursing educators and healthcare organizations to better determine how to cultivate and improve PS competence in undergraduate nursing students by establishing documented policies or by improving intervention efficacy.

## Data Availability

The datasets used and/or analysed during the current study are available from the corresponding author on reasonable request.

## Abbreviations

H-PEPSS: the Health Professional Education in Patient Safety Survey
PS: Patient safety

## Footnotes

1 Hospitals in China are organized according to a 3-tier system that recognizes a hospital’s ability to provide medical care, medical education, and conduct medical research. Based on this, hospitals are designated as Primary, Secondary or Tertiary institutions.
